# Editorial: Biomarker-driven strategies for personalized management of systemic inflammatory response syndrome

**DOI:** 10.3389/fimmu.2026.1820314

**Published:** 2026-03-13

**Authors:** Sara Bobillo-Perez, Iolanda Jordan, Bernhard Ryffel

**Affiliations:** 1 Pediatric Intensive Care Unit, Hospital Sant Joan de Déu, Universitat de Barcelona, Barcelona, Spain; 2 Universitat de Barcelona (UB), Barcelona, Spain; 3 INEM UMR7355 University of Orleans, Orleans, France

**Keywords:** biomarker, inflammatory heterogeneity, multi-omic analyses, personalized management, SIRS

Systemic inflammatory response syndrome (SIRS) encompasses diverse inflammatory insults, from sepsis and trauma to autoinflammatory conditions, that lead to life-threatening organ dysfunction. Current diagnostic and therapeutic paradigms fail to address the heterogeneity of these patients. However, biomarkers promise precision by allowing early differentiation of etiologies, dynamic risk assessment, and tailored immunomodulation across patient populations. This Research Topic in *Frontiers in Immunology* presents 11 articles that advance these aims through mechanistic dissection, clinical translation, and ethical frameworks, redefining SIRS as an orchestrated immunological state.

The contributions follow two main goals: SIRS pathobiology and biomarker-AI integrated models. A group of these articles focuses on pathobiology via multi-omics signatures, mapping how immune activation leads to organ resolution across different etiologies and ages. The other focus is the ‘how-to’ of implementing biomarkers through artificial intelligence (AI) integration, targeted interventions, and governance models to ensure these tools are deployed equitably, especially in pediatric emergencies, and address implementation barriers.

Preclinical investigations establish core drivers. Jin et al. reveal liver-initiated propagation to multi-organ failure in a porcine cytokine storm model, identifying organ-specific biomarkers for interception. Chekroune et al. demonstrate disrupted macrophage autophagy driving cell death and iron dysregulation in a mouse lipopolysaccharide-induced inflammation model, positioning autophagic modulation as a therapeutic axis. These models show varied mechanisms and favor combined biomarker panels over single tests.

Human-focused studies pioneer accessible diagnostics and interventions. Sallard et al. uncover saliva’s type-dependent antiviral activity against adenoviruses, strongly inhibiting species B while enhancing species D/E infectivity, which supports its broader role in mucosal immune responses and enables non-invasive surveillance of infection-triggered cases. Lalla et al. link hyperoxia exposure to septic shock mortality, proposing biomarker-calibrated oxygenation limits. Necipoglu et al. validates saliva-serum cytokine profiling for prognostication in pediatric community-acquired pneumonia. Bai et al. document bortezomib resolving refractory hyperinflammation in Schnitzler syndrome. Another publication (Coletti Giesler) tracks responses to low-pressure hyperbaric oxygen in chronic inflammatory states, reducing key inflammatory biomarkers. They highlight its potential in innate immune modulation. From a different perspective, Giri et al. study shows the systemic immune-inflammation index (SII), calculated simply from routine blood counts (platelets × neutrophils ÷ lymphocytes), predicts higher death risk in critically ill COPD patients with SIRS. This cheap, accessible biomarker enables immediate risk stratification for ICU decisions in any hospital.

Other works link to neurology and immunity. The review included (Armstrong et al.) identifies PRR hyperactivation as the central trigger of cytokine storms in severe infections like sepsis and COVID-19, and exposes PRR pathway modulation as a promising therapeutic target to restore immune homeostasis and prevent systemic collapse. Tian et al. examines neurological sequelae through cytokine networks, bridging central and peripheral responses.

Ultimately, scientific advancement must be paired with ethical responsibility. Stolte et al. explore this through expert interviews, focusing on the unique challenges of implementing OMICS and AI-driven therapies in pediatric SIRS. Their findings call for adaptive consent processes using visual tools for child empowerment, alongside pragmatic emergency protocols and interdisciplinary guidelines that move beyond simple precautionary restrictions. Experts further emphasize data stewardship, equity safeguards against multi-omics biases, and the need for specialized training to ensure trustworthy AI systems during clinical crises.

Together, these 11 articles provide a comprehensive roadmap for personalizing SIRS care, bridging the gap between accessible diagnostics, like saliva tests and simple blood indices, and the complex ethical frameworks required for their use. By highlighting the risks of data bias and clinical inequality, these contributors call for a shift toward globally coordinated trials. The goal is clear: to move away from empirical, ‘blind’ treatments in favour of biomarker-guided strategies that prioritize both clinical efficacy and social equity.

Collectively, these contributions move beyond isolated markers by integrating diverse tools, from non-invasive saliva proxies to high-fidelity animal models, into cohesive clinical platforms. Ultimately, this Research Topic serves as a catalyst, guiding SIRS management toward a more accountable and truly patient-centered future.

